# Role of hemagglutinin esterase protein in neurological manifestation of COVID-19

**DOI:** 10.1186/s12987-021-00271-2

**Published:** 2021-08-16

**Authors:** Milad Zandi, Hassan Karami, Saber Soltani

**Affiliations:** Department of Virology, School of Public Health, Tehran University of Medical Sciences, Tehran, Iran

Dear editor,

We read with interest an article by McQuaid et al. [1], in this article, the authors stated that “The lipid plasma membrane has structural proteins, namely the spike protein (SP), membrane protein, small membrane protein and hemagglutinin-esterase” [1]. For this claim, the authors cited a related study, but there is not any evidence on hemagglutinin-esterase gene in that cited reference [2]. However, according to the scientific evidence the genome of SARS-CoV-2 lacks the hemagglutinin-esterase (HE) gene and it has no HE glycoprotein [3–6], thus HE has no role in neurological manifestation of COVID-19.

From the emergence of SARS-CoV-2 in December 2019, a great amount of efforts and research have been made to characterize the virus and the clinical course of coronavirus disease 2019 (COVID-19) [7]. SARS-CoV-2 as an enveloped, positive-strand RNA virus is a member of *Coronaviridae* family in *Nidovirales* order [8]. According the genetic properties *Coronaviridae* family is subdivided to 4 genera including: alpha, beta, gamma and deltacoronavirus [9]. In addition, betacoronavirus genus has five subgenera: *Embecovirus* (lineage A), *Sarbecovirus* (lineage B), *Merbecovirus* (lineage C), *Nobecovirus* (lineage D) and *Hibecovirus *[10].

Between human coronaviruses, OC43-CoV and HKU1-CoV are considered as betacoronaviruses of linage A, however, both SARS-CoV and SARS-CoV-2 are betacoronaviruses of linage B and also MERS-CoV is a member of betacoronavirus of C lineage [11].

Between coronaviruses, betacoronaviruses lineage A including: bovine-CoV, OC43-CoV, HKU1-CoV, mouse hepatitis virus(MHV), harbor HE which acts functionally like spike (S) protein [12, 13]. The gene of HE is transmitted from influenza virus C/D to betacoronavirus lineage A progenitor via horizontal gene as 9-*O*-Ac-SA–specific hemagglutinin-esterase-fusion (HEF) [11]. The HE in betacoronaviruses lineage A does not show activity of membrane fusion unlike HEF in influenza virus C/D [11]. SARS-CoV-2 as a betacoronavirus of *Sarbecovirus* subgenus comprises four structural proteins spike (S), envelope (E), membrane protein (M), and nucleoprotein (N) and lacks HE [6].

In conclusion, HE gene is absent in betacoronaviruses such as SARS-CoV-2, MERS-CoV and SARS-CoV, thus HE has no role in neurological manifestation of COVID-19, however, some betacoronaviruses in subgenera *Embecovirus* have HE.

## Data Availability

Not applicable.

## Abbreviations

COVID-19: Coronavirus Disease 2019
SARS-CoV: Severe acute respiratory syndrome-CoV
SARS-CoV-2: Severe acute respiratory syndrome-CoV-2
MERS-CoV: Middle East Respiratory Syndrome CoV
